# Modeling indicates degradation of mRNA and protein as a potential regulation mechanisms during cold acclimation

**DOI:** 10.1007/s10265-021-01294-4

**Published:** 2021-04-23

**Authors:** Maria Krantz, Julia Legen, Yang Gao, Reimo Zoschke, Christian Schmitz-Linneweber, Edda Klipp

**Affiliations:** 1 Theoretical Biophysics, Institute of Biology, Faculty of Life Sciences, Humboldt-Universität zu Berlin, 10099 Berlin, Germany; 2 Molecular Genetics, Institute of Biology, Faculty of Life Sciences, Humboldt-Universität zu Berlin, 10099 Berlin, Germany; 3 Translational Regulation in Plants, Max Planck Institute of Moleculare Plant Physiology, 14476 Potsdam, Germany

**Keywords:** Acclimation, Chloroplast, Modeling, Temperature, Transcription, Translation

## Abstract

Plants are constantly exposed to temperature fluctuations, which have direct effects on all cellular reactions because temperature influences reaction likelihood and speed. Chloroplasts are crucial to temperature acclimation responses of plants, due to their photosynthetic reactions whose products play a central role in plant metabolism. Consequently, chloroplasts serve as sensors of temperature changes and are simultaneously major targets of temperature acclimation. The core subunits of the complexes involved in the light reactions of photosynthesis are encoded in the chloroplast. As a result, it is assumed that temperature acclimation in plants requires regulatory responses in chloroplast gene expression and protein turnover. We conducted western blot experiments to assess changes in the accumulation of two photosynthetic complexes (PSII, and Cytb6f complex) and the ATP synthase in tobacco plants over two days of acclimation to low temperature. Surprisingly, the concentration of proteins within the chloroplast varied negligibly compared to controls. To explain this observation, we used a simplified Ordinary Differential Equation (ODE) model of transcription, translation, mRNA degradation and protein degradation to explain how the protein concentration can be kept constant. This model takes into account temperature effects on these processes. Through simulations of the ODE model, we show that mRNA and protein degradation are possible targets for control during temperature acclimation. Our model provides a basis for future directions in research and the analysis of future results.

**Supplementary Information:**

The online version contains supplementary material available at 10.1007/s10265-021-01294-4.

## Introduction

Cold-acclimation describes the process by which organisms adjust their metabolism and other cellular processes to low temperatures. It refers to the adjustment of cellular processes to low, but non-freezing temperatures. Temperature acclimation as a short-term response has received less attention in modelling approaches than medium to long-term reactions that are usually referred to as adaptation processes (Somero and Hochachka 1971)⁠. The chloroplast is a central point in this acclimation process (Crosatti et al. 2013)⁠⁠⁠⁠. Chloroplasts can act as sensors for low temperature and are able to activate specific signaling pathways to trigger cold acclimation (Kleine et al. 2020)⁠⁠. The chloroplast itself also undergoes cold acclimation by adjusting its metabolism, signaling and translation machinery (Crosatti et al. 2013; Leister et al. 2017)⁠⁠⁠. This is necessary to ensure that the chloroplast can fulfill its function in the plant cell, performing photosynthesis and delivering energy to the cell, even under cold temperature stress (Crosatti et al. 2013)⁠.

Changes in metabolism and gene expression of the whole cell have been extensively studied and also the specific adjustment of metabolism and signaling of the chloroplast in reaction to low temperature has been investigated (Fürtauer et al. 2019; Guy et al. 1985; Herrmann et al. 2019; Leister et al. 2017; Lissarre et al. 2010; Liu et al. 2018)⁠⁠. Especially changes in the photosynthetic apparatus have been extensively investigated (Ensminger et al. 2006; Janmohammadi et al. 2015)⁠, showing that cells undergo specific changes to adjust their metabolism and signaling to low temperatures. However, the role of the chloroplast in the control of cold-acclimation of plants is only now emerging.

An important aspect in studying temperature-associated changes in cells and their behavior are thermodynamic effects. Catalytic reactions are known to become slower at lower temperatures and this effect is well understood. The Van’t Hoff rule states that a reaction becomes 2–3 times slower for each 10 K in temperature drop. Another well-known effect is the rigidification of membranes, which influences numerous processes in the cell. However, these are not the only effects. As temperature drops, also the movement of all molecules becomes slower, because the diffusion coefficient is also dependent on temperature. Especially for high-order reactions like transcription or translation, where many molecules need to encounter one another, the effect of a slower diffusion and therefore a lower encounter rate will be significant.

In the work presented here, we examined how the lower encounter rate due to lower diffusion coefficients at lower temperatures influences transcription and translation as well as degradation of both mRNAs and proteins. Using protein data obtained from chloroplasts of tobacco plants during cold acclimation we developed a model of translation and transcription under lower temperatures and suggest possible strategies the chloroplast could use to stabilize protein concentration in low temperatures.

## Material and methods

### Experimental procedures

For the western blot analysis, three week old wild-type tobacco plants were used. Tobacco seeds were germinated in day-night cycles of 16 h light (100 µmol m^−2^ s^−1^)/22 °C and 8 h dark/22 °C with 75% humidity. Eight days after sowing, seedlings were transplanted to individual pots. The plants were grown in the same condition for additional two days and then transferred to control conditions with day-night cycles of 16 h light (350 µmol m^−2^ s^−1^)/24 °C with 60% humidity and 8 h dark/20 °C with 55% humidity. Five hours after the start of the photoperiod 21 days after sowing, the plants were shifted to low temperature (12 °C). At 0 min, 0.5 min, 5 min, 20 min, 1 h, 5 h, 1 day and 2 days after the temperature shift the aerial part of the plants was harvested and a total protein extract was prepared after the method presented in (Tsugama et al. 2011)⁠⁠. Western blot analysis was performed for the three proteins PetA, AtpA and PsbA for each of the time points for one shifted plant and a non-shifted control plant of the same age. The western Blots were quantified using the Image Lab Software from Bio-Rad and the relative changes of the protein concentration were calculated compared to the concentration at 0 min. For each time point three sample replicates were performed and the average change in protein accumulation was calculated.

### Mathematical modeling

For the mathematical modeling, we suggest a simple transcription-translation model. It encompasses a transcription reaction, an mRNA degradation reaction, a translation reaction, and a protein degradation reaction. The model consists of a set of ordinary differential equations (ODEs) for the changes in rates and concentrations following temperature shifts to lower temperatures. The four reactions represent the more complex set of events happening during transcription, translation and degradation and are, therefore, simplified lumped reactions.

To obtain insight into the change in translation rate, the measurements from (Bottomley et al. 1971)⁠ were used. Analyzing these data led to a change in translation rate of approximately 2.5 fold per 10 K, which was used as a relative change of the translation rate for the model. The role of temperature in the dynamics and steady states of chemical reaction systems is considered by application of the Arrhenius equation

1 $$ k = A \cdot e^{{\frac{ - \Delta G}{{RT}}}} $$

where *k* is the rate constant, *A* is a factor depending on the collision rate, *T* is the absolute temperature in Kelvin and *R* is the universal gas constant.

Values for *ΔG* and *A* are tabulated for many basic chemical and biochemical reactions, but they are not available for the complex reactions examined in this paper. Therefore, we have chosen an alternative approach and related the temperature-dependent change of the reaction rate *k* to the estimated number of encounters between two molecules required for each complex reaction to happen. This is based on the fact that the diffusion coefficient D in liquids depends on temperature according to the Einstein-Stokes equation

2 $$ D = \frac{{k_{b} T}}{{6\pi \eta R_{0} }} $$

with k_b_ being the Boltzmann constant, T being the Temperature, η being the dynamic viscosity of the solvent and R_0_ being the hydrodynamic radius of the particle (Einstein 1905)⁠⁠⁠.

The rate of a reaction, therefore, depends on temperature in two different ways. First, the encounter rate (and therefore the A in the Arrhenius equation) depends on temperature due to the temperature dependence of diffusion. Second, the number of collisions that lead to a reaction depends on temperature, which is given by the exponential term of the Arrhenius equation.

The models were compiled and simulated using the stimator package in python2.7 and visualized using the matplotlib package for python.

The python file containing the estimator code as well as the ODEs derived from the reactions can be found as Online Resource. The model reactions are:

$$ {\text{mRNA production}}:\, \rightarrow {\text{mRNA}},\; {\text{rate}} = {\text{k}}_{1} $$

$$ {\text{Protein production}}:\, \rightarrow {\text{Protein}},{\text{ rate}} = {\text{ k}}_{{3}} \times {\text{mRNA}} $$

$$ {\text{mRNA degradation}}:{\text{ mRNA}} \rightarrow ,\, {\text{rate}} = {\text{ k}}_{{2}} \times {\text{mRNA}} $$

$$ {\text{Protein degradation}}:{\text{ Protein}} \rightarrow ,\, {\text{rate}} = {\text{ k}}_{{4}} \times {\text{Protein}} $$

The corresponding values for the rate constants can be found in Tables 1, 2. Each simulation was started with none of the species being present and run until a steady state was reached. In the steady state, the rates were changed according to Table 1 to simulate the temperature decrease.

**Table 1:** Rate constants of the temperature-dependent model at different temperatures

Rate		Value at 24 °C [a.u.]	Value at 14 °C [a.u.]
k_1_	Transcription	2.5	0.25
k_2_	Degradation of mRNA	1	0.5
k_3_	Translation	2.5	0.25
k_4_	Degradation of Protein	1	0.5

**Table 2:** Rate constants used for the simulation shown in Fig. 3 after temperature shift

Rate	Simulation 1 (Fig. 3a)	Simulation 2 (Fig. 3b)	Simulation 3 (Fig. 3c)	Simulation 4 (Fig. 3d)
k_1_	0.25	0.25	0.25	0.25
k_2_	0.1	0.5	0.1	0.5
k_3_	0.25	0.25	0.25	0.25
k_4_	0.1	0.5	0.5	0.1

The model was simulated until it reached steady state with the parameter values shown in Table 1 for a temperature of 24 °C and were then changed to the values shown here

## Results

### Western blot analysis

**Fig. 1:**
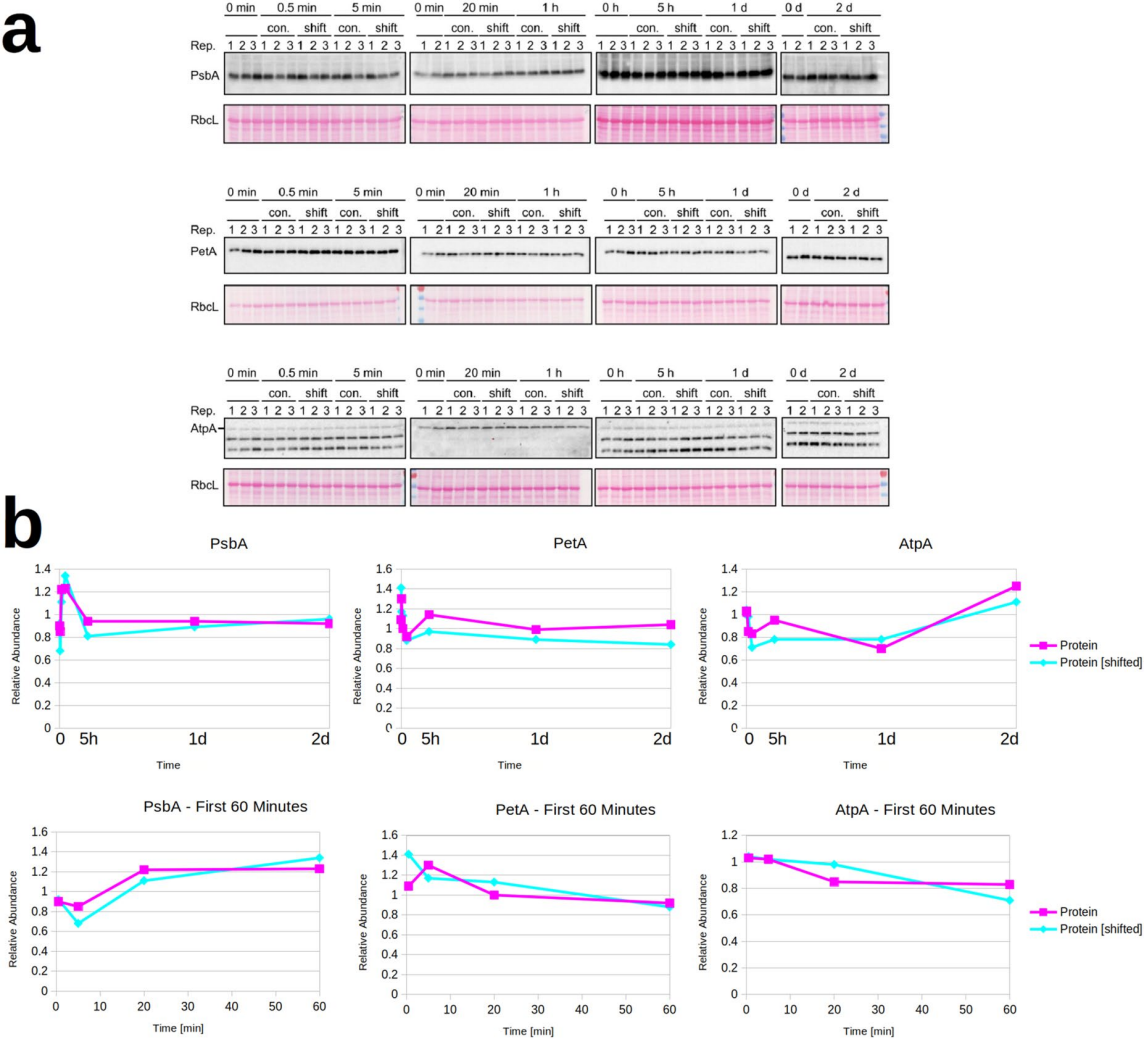
Western Blots of PsbA, PetA and AtpA from whole-cell lysates of tobacco plants. **a** shows the Western Blots. Tissue weight was used as a loading control. The plants were shifted from 24 °C to 12 °C and plants were harvested at 30 s, 5 min, 20 min, 1 h, 5 h, 1 day and 2 days and compared to the control plants, which were grown in 24 °C and harvested at the same time. Below in **b** are the plots of the quantification of the western blots. The time course for the protein in the control plant (pink) and the plants shifted to 12 °C (light blue) are shown

Analysis of the western blots revealed, that for the three analyzed proteins PetA, PsbA and AtpA, there was a minor change in concentration compared to the control at 0 min, but the proteins from the shifted plants and the control plants showed the same dynamic for all time points in the time series (Fig. 1). This means that there was no or very little difference in protein amount between the control plants, which were kept at 24 °C, and the plants shifted to 12 °C. As can be seen from the plots of the quantified western blots (Fig. 1b), the protein concentrations show some alterations, possibly due to circadian rhythms and/or plant development. As the time points for the measurements were denser in the beginning, it is likely that these fluctuations are only visible at the beginning because the later time points were too sparse to catch them. PetA, PsbA and AtpA are core subunits of the cytochrome b_6_f complex, the photosystem II and the ATP synthase, respectively, which do not accumulate to substantial amounts outside of their complexes, and can therefore also be seen as reporters of complex accumulation (Barkan 1993)⁠⁠⁠. The cytochrome b_6_f complex, the photosystem II and the ATP synthase are three of the four complexes which carry out the light reactions of photosynthesis and ATP production in the chloroplast. They are embedded in the thylakoid membrane.

### Description of the temperature-dependent model

**Fig. 2:**
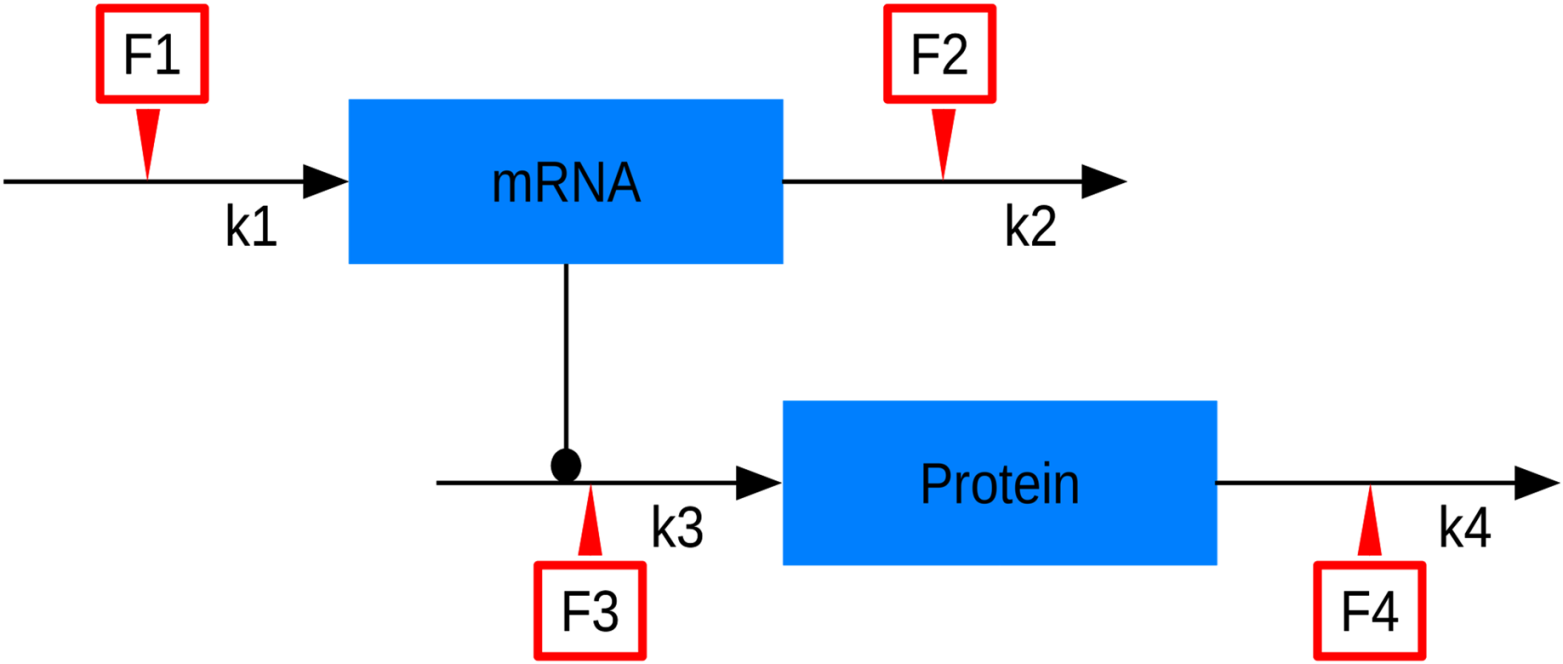
Graphical representation of the ODE model. It consists of transcription, translation and two degradation reactions. Red arrows indicate possible control points by the cell. Values for the rates k_1_ to k_4_ are given in Table 1. F1 to F4 indicate the possible effects of temperature as well as possible intervention points by the chloroplast

To understand the effects of temperature changes on protein concentration, we formulated a model of transcription and translation as well as mRNA degradation and protein degradation (Fig. 2, Table1). For constant temperature, temporal simulations of the model from arbitrary initial states run into a steady state of mRNA and protein concentration. These steady states are given by

3 $$ mRNA^{SS} = \frac{{k_{1} }}{{k_{2} }} $$

for the mRNA and

4 $$ Protein^{SS} = \frac{{k_{1} \cdot k_{3} }}{{k_{2} \cdot k_{4} }} $$

for the protein.

**Fig. 3:**
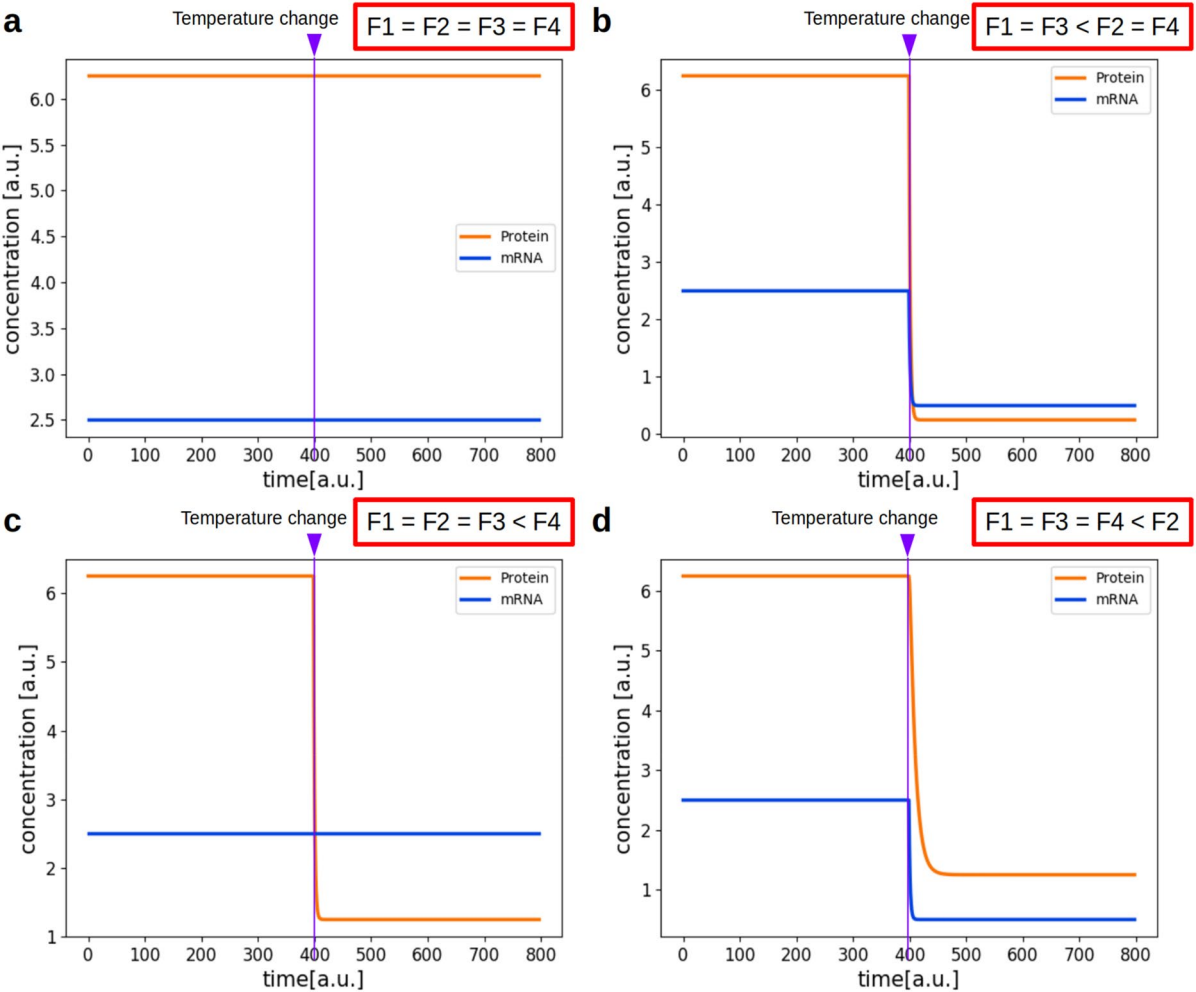
Simulation of the model with temperature considerations. F1 to F4 in the red boxes correspond to the red boxes in Fig. 2 and denote the temperature factor which effects the corresponding reactions. **a** All reactions are affected by the same factor after temperature change. **b** Translation and transcription are affected by the same factor, while the two degradation reactions are affected by a smaller factor, therefore the degradation is less affected by the temperature change and can proceed at a higher rate compared to translation and transcription. **c** Only the degradation of the protein is affected by a smaller factor. **d** Only the degradation of the mRNA is affected by a smaller factor. See Table 2 for rates used in the simulations.

To understand the observation of constant protein concentration after temperature shift and, hence, the effects of temperature change on translation and transcription as well as on the steady state values of mRNA and protein, we first wanted to identify the relevant changes in the rates as expressed in Eq. (1). Since transcription and translation are composite processes, also the temperature effects on these reactions are very complex. Therefore, we aimed at identifying a factor that quantifies these effects in a simple manner. We first tested a case in which all reactions were affected by the same factor due to the shift to lower temperature (Fig. 3a). In this case, protein and mRNA concentration stay constant, because the rate constants for transcription (*k*_1_), translation (*k*_3_) and for the two degradation reactions (*k*_2_ and *k*_4_) are equally affected by the change in temperature. This would explain the experimental data shown in Fig. 1. But is this a biologically likely scenario?

The effect of temperature on reactions like transcription, translation and degradation is determined by the Arrhenius equation given in Eq. (1). However, this would require measurements of activation energy for these reactions. Since these values are not available, an approximation is used, which does not quantify the direct effect of temperature on the reactions, but assesses the difference in effect between them. A major determinant for the speed of biological reactions is the rate at which two molecules encounter each other to make a reaction happen, which will be termed the encounter rate here. The encounter rate depends on the concentration of the reaction partners and their diffusion rate. The diffusion rate itself depends on temperature. A second factor which majorly determines the speed of a reaction is the fraction of encounters which lead to a reaction. This fraction also depends on temperature. These two factors are used as an approximation to assess the differential effect that a decrease in temperature will have on transcription and translation on the one side and degradation of mRNA and protein on the other side.

For composite processes like translation and transcription, the encounter rate and the fraction of encounters which lead to a reaction are substantial factors in reaction speed, since a large number of encounters is necessary to form an mRNA molecule or a protein. To get a semi-quantitative understanding of the regulation principles following temperature change, we will provide rough estimates that illustrate the underlying principles.

First, we assume a protein of 350 amino acids as an example. To synthesize the corresponding mRNA, at least three times as many nucleotides are needed, not considering splicing or any other modifications of the mRNA. That means that the number of necessary successful encounters for making a molecule of mRNA is in the order of magnitude of 1000. For translation, this number of encounters between the ribosome and the loaded tRNAs is roughly the number of amino acids, but the corresponding tRNAs need to be loaded first. Therefore, the encounter rate for translation is approximately in the same order of magnitude as for transcription. In these considerations we did not consider the possibility of encountering the wrong nucleotide or a tRNA loaded with the wrong amino acid.

Second, degradation is also a complex and multifaceted process. Many different proteases exist in chloroplasts and they seem to have distinct target proteins and functions. However, the interesting aspect for our considerations is the encounter rate. A protease needs one encounter for a protein, which has been targeted for degradation. The entry of the protein seems to be ATP-dependent, however the degradation seems to be ATP-independent. We therefore assume the encounter rate to be lower for degradation than for translation and transcription. A similar assumption can be made for mRNA degradation.

Based on this argumentation, we assume that temperature affects degradation on one side and transcription and translation on the other side differently. Since more molecules need to encounter one another in transcription and translation than in the two degradation reactions, transcription (k_1_) and translation (k_3_) should be affected by a decrease in temperature to a higher extent. Since degradation (k_2_ and k_4_) requires less molecules to encounter one another, the effect of a decrease in temperature should be smaller than for transcription (k_1_) and translation (k_3_). We therefore assume that the degradation reactions are affected to a lesser degree by the shift towards colder temperatures. This is expressed in the model by altering the rates of the corresponding reactions. Translation and transcription are affected by the shift in temperature to a higher degree, therefore their rates (k_1_ and k_3_) are decreased by a higher factor than the rates for the degradation reactions (k_2_ and k_4_). Thus, we do not focus on how much the rates decrease due to the temperature shift, but on the difference in decrease between the reactions involved in the production of macromolecules (k_1_ and k_3_) and the reactions involved in their degradation (k_2_ and k_4_).

On this basis, we simulated the model again, this time using the rates given in Table 1 for 14 °C. For these rates we take into consideration that, while translation (k_3_) and transcription (k_1_) are affected by a factor of 10 for a temperature increase of 10 °C, the two degradation rates (k_2_ and k_4_) are only affected by a factor of 2. This is due to the assumptions discussed above, in which the number of necessary encounters is much higher for translation and transcription than for degradation. The resulting time course is shown in Fig. 3b. Here, we assume a temperature drop from 24 °C to 14 °C at time *t* = 400 and an immediate change of all rates. Both mRNA and protein concentration decrease after the temperature change because translation (k_3_) and transcription (k_1_) become slower to a greater extent than degradation (k_2_ and k_4_). As an alternative scenario, Fig. 3c shows the time course when only protein degradation (k_4_) is affected by a factor of 2, while mRNA degradation (k_2_) is affected by a factor 10. Figure 3d shows the reverse case, where protein degradation (k_4_) is lowered tenfold and mRNA degradation (k_2_) twofold. The rates used for these simulations can be found in Table 2. 

### Analysis of possible control points

**Fig. 4:**
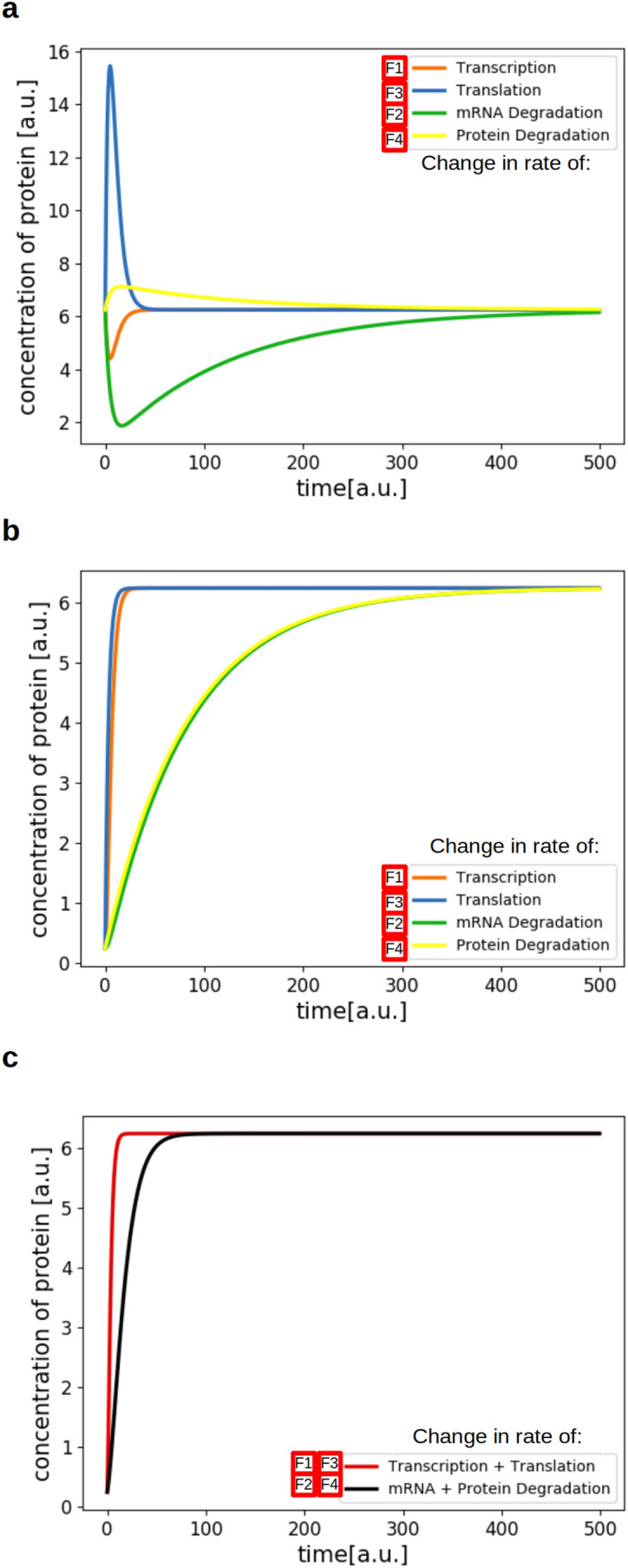
Simulation of the model with rates changed to return to the steady state before temperature shift. The rates needed to return to the steady state the system had before the temperature change were calculated according to Eq. (2). The colors indicate which rate was changed. For **a**, the starting point of the simulation is the steady state before the temperature shift, representing an immediate response by the cell. For **b**, the starting point of the simulation was the steady state of the system after temperature shift, showing the behavior if the cell first settles into the new steady state. For the simulation in **c**, both transcription and translation were upregulated fivefold or the degradation of mRNA and protein were downregulated fivefold, starting from the steady state after temperature shift

As we have discussed above, it is likely that transcription and translation are differently affected by a temperature change than degradation. One can, therefore, expect that the cell needs to counteract the effects of lower temperature to keep protein concentrations constant. Within the frame of our model, there are four opportunities for control (which are referenced in Fig. 2 as F1 to F4), i.e., transcription (F1), translation (F3), mRNA degradation (F2) or protein degradation (F4), or combinations thereof. These potential control points are indicated in Fig. 2 as red arrows. Given the steady states in Eqs. (3) and (4), we can calculate the exact values, which the corresponding rate constants have to reach in order to stabilize the protein concentration and to return to exactly the steady state before the temperature shift. Using the values for the rates from Table 1 at 14 °C, we can calculate how much rates have to change. If only one of the rate can adapt, then the rates for either transcription (F1) or translation (F3) would have to increase 25 fold, while the rates for degradation of either mRNA or protein would have to decrease 25 fold. This would lead to a return to the same steady state the system had before the temperature shift (Fig. 4). These values depend on the change in rates due to temperature change. The 25-fold increase or decrease here results from the specific combination of rates chosen for this case. When choosing another factor for the change in the rates due to temperature change, the values can be calculated using the steady state equations.

We also took into consideration that the system can either immediately react to the temperature change (Fig. 4a) or first settle into the new steady state at the lower temperature and then counterbalance by a regulation measure (Fig. 4b). In case of the direct change of the corresponding rate, before the system settles into the new steady state, the upregulation of translation (F3) and the downregulation of protein degradation (F4) would both lead to a short overshoot in protein concentration before the system settles back into the steady state. An upregulation of transcription (F1) and the downregulation of mRNA degradation (F2) would lead to a drop in protein concentration before the steady state is restored. If the system first settles into the new steady state at the lower temperature, the 25-fold increase of transcription (F1) or translation (F3) would rapidly return the system to the steady state before temperature shift, while the 25-fold decrease of mRNA and protein degradation (F2 and F4) would return the system to steady state much slower. Thus, we note a difference in the response time to the temperature signal.

It is also worth noting that the 25-fold increase of the transcription rate (F1) would lead to a more than 4 times higher mRNA concentration than before temperature shift. This would be necessary to stabilize the protein concentration during cold shift when only using the transcription as a control point. The same applies for mRNA degradation (F2). When only the rate of mRNA degradation is used as a control point, the concentration of mRNA would have to rise to more than 4 times the concentration before temperature shift to stabilize the protein concentration.

The 25-fold reduction of degradation rates for either mRNA or protein (F2 and F4) would also let the system settle back into the steady state of protein concentration before the temperature shift. As can be seen in Fig. 4, this return to steady state is much slower compared to the upregulation of transcription or translation (F1 and F3). However, these rates were calculated so that the system would settle into the steady state. Alternatively, it would be possible to reach the desired protein concentration faster by decreasing the degradation rate further, e.g. complete short-term stop of degradation. This would not lead to the same steady state, but it would be possible to have a higher decrease in degradation rate for a shorter time and to then increase the degradation rate again to reach the same protein concentration as before the temperature shift.

As another scenario, it is also possible that the chloroplast uses a combination of these control points. Figure 4c shows two of the potential combinations, i.e., the combination of transcription and translation (F1 and F3) with a fivefold increase each as well as the combination of mRNA and protein degradation (F2 and F4) with a fivefold decrease for both. By combining two control points, each of the factors can be smaller than when only one control point is used. In the case of transcription and translation, each of them only needs to be upregulated such that their product of fold-changes is 25-fold, likewise for the combination of mRNA and protein degradation, where the production of fold-changes downwards must equal 25-fold.

A combination of regulating degradation and production would also be possible. To reach the steady state, the producing and degrading reactions need to be in a specified ratio to one another. The time it takes the system to move from one steady state to another is referred to as transition time. As can be seen in Fig. 4, the transition times τ are different for the regulation of translation, transcription and degradation, the two degradation regulation points being much slower. The transition times can be calculated as

5 $$ \tau_{mRNA} = \frac{1}{{k_{1} + k_{2} }} $$

for the case of mRNA regulation and

6 $$ \tau_{Protein} = \frac{1}{{k_{1} + k_{2} + k_{3} + k_{4} }} $$

for the case of protein regulation.

**Fig. 5:**
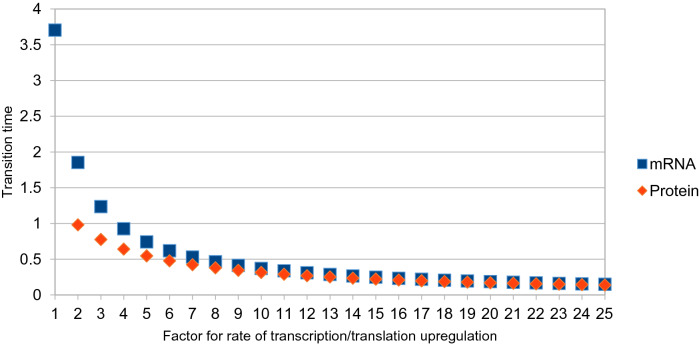
Transition time depending on the rates of translation or transcription and degradation. For the simulations shown in Fig. 4a, c, the rates of transcription and mRNA degradation were altered in a way, that the resulting steady state was the same as in Fig. 4. This means that the ratio between the factor by which transcription is upregulated and the factor by which mRNA degradation is downregulated was always 25. The transition time is defined as τ_mRNA_ = 1/(k_1_ + k_2_). The same was done for translation and protein degradation. Here the transition time is defined as τ_Protein_ = 1/(k_1_ + k_2_ + k_3_ + k_4_). The rate by which translation and transcription are upregulated can be seen as an estimate for the increase in ATP consumption for these processes

As we saw above, to reach the steady state, transcription needs to be upregulated 25-fold or mRNA degradation needs to be downregulated 25-fold. Controlling transcription and mRNA degradation at the same time means that, in order to reach the steady state, the rates for transcription and degradation need to have a defined constant ratio to one another. The same holds for translation and protein degradation. Using the formulas for τ given in Eqs. (5) and (6), we can calculate the transition time and plot it against the factor, by which the transcription or translation need to be upregulated and against the factor by which the corresponding degradation reactions need to be downregulated (Fig. 5). In this case, this is only done for either mRNA or protein as the control point. Because the factor by which the transcription and translation are upregulated is proportional to the increase in costs for these two processes, the factor can be seen as a measure for costs associated with the corresponding regulation. Regulating only transcription or translation is faster, but costs the cell more, while also regulating the degradation is slower, but more cost efficient. Therefore, Fig. 5 presents a Pareto front of the system for the case that both low costs and minimal transition times are considered as objective functions for the adaptation to the drop in temperature.

### Comparison to western blots

Comparing our computational results to the quantification of the western blots (Fig. 1) shows that we were able to reproduce the principal behavior observed in the experiments. The western blots show no or very little difference between the control and the temperature shifted plants. This is reproduced in our simulations. The remaining question is how the chloroplast would achieve the stabilization of protein concentration after the temperature shift. The most intriguing protein in this consideration is possibly PsbA, as it is one of the two core subunits of the Photosystem II, the D1 subunit. As such, it is subjected to damage by light and therefore needs to be replaced constantly, thereby causing a high turnover of the PsbA protein (Järvi et al. 2015)⁠⁠. It can be assumed that the PsbA protein also needs to be replaced under colder conditions and therefore, for this protein, it is likely that the regulation happens by upregulating transcription or translation. As for the other two proteins, PetA and AtpA, it is possible that they are either regulated by downregulating their degradation or upregulating transcription and translation.

## Discussion

Cold acclimation is an important process in plants. Especially the chloroplast needs to adapt to lower temperatures to maintain its photosynthetic ability (Crosatti et al. 2013)⁠⁠. The processes of cold acclimation are subject to current research and many new strategies have recently been unraveled (Herrmann et al. 2019)⁠⁠⁠⁠.

Our western blots of three photosynthetic core subunits showed that the protein concentration remains constant after temperature shift (Fig. 1). We decided to use a model of differential equations to dissect potential regulation mechanisms the cell could use to accomplish this result. The model is a simplistic representation of protein production, encompassing four reactions: transcription, translation, mRNA degradation and protein degradation. With this model, we could simulate the behavior of the system and make predictions about possible regulations.

Our first observation is that the concentrations of mRNA and protein would not change after a cold-shift, when the producing and degrading reactions are affected to the same degree (Fig. 2a). This could be a possible explanation for the concentrations observed in the western blots. However, it is unlikely that transcription and translation on the one hand and degradation on the other hand are affected to the same degree for the three analyzed proteins by a cold shift, since the encounter rates of these reactions are very different. It is therefore likely that degradation is less affected by cold shift than translation and transcription. This would lead to a decline in mRNA and protein concentrations (Fig. 2b).

The chloroplast needs to adjust to these conditions to keep the concentration of important proteins constant. To achieve this, it has four principle targets. It could upregulate either transcription or translation or it could down regulate the degradation of either mRNA or protein (see Fig. 2). We simulated these four possibilities (Fig. 4). Through these simulations, we made some general observations. First, in case of regulation of transcription to counteract cold effects, the mRNA concentration would have to increase to a four times higher level than before the cold shock to restore the protein concentration to pre-cold shift temperatures. This would mean that the transcription rate needs to increase 25-fold. As discussed above, lower temperature would lead to a substantial decrease in transcription due to a lower encounter rate. It is therefore unlikely that the cell would be able to simultaneously attain such an increase in transcription rate, since all processes generally become slower and more transcription machinery would have to be available to achieve this upregulation. Another option would be to increase the translation rate. To stabilize the protein concentration the translation rate would have to increase about 25-fold compared to the rate after cold shift. The same considerations as for transcription also apply here. The cell would have to be able to recruit a considerable number of ribosomes to upregulate translation sufficiently. Because translation will be slower in lower temperatures it is unlikely that the cell will manage to considerably upregulate the translation of most proteins. This would only be possible if ribosomes are recruited from other mRNAs, thereby downregulating these genes. Whether the cell is able to upregulate transcription and translation sufficiently under cold stress to stabilize the protein concentration is an open question. It is unlikely that it would be possible to quickly produce more polymerases, nucleotides, ribosomes and amino acids, as these processes are subject to the same cold-induced restrictions that were discussed earlier. As we saw when calculating the necessary rates for transcription and translation the rates would have to increase 25-fold, compared to what we assumed for the cold-shifted plants. It would, however, be possible to limit the space these molecules have and therefore make encounters between them more likely. The cell or the chloroplast could achieve this by compartmentalization or by using proteins to bind for example ribosomes, mRNAs and tRNAs and bring them in proximity to one another to increase the encounter rate.

The cell could also control the other side of these processes, the mRNA and protein degradation. To stabilize the mRNA concentration, its degradation rate would have to be reduced fivefold, while the rate of protein degradation would have to be 25 times lower than the rate we assumed for lower temperature, as can be calculated from the equations for the steady state of mRNA and protein given in Eqs. (3) and (4). This regulation would be easier to achieve than an upregulation of transcription or translation at lower temperature. Degradation is known to play an important role for regulation of mRNA levels in the chloroplast (Leister et al. 2017)⁠⁠ and it is, therefore, not unlikely that this regulation is also used in the case of adjustments after cold shift to stabilize mRNA and, possibly, also protein levels. We, therefore, suggest a model in which the chloroplast uses regulation of the proteases after cold shift to stabilize the protein concentration. It is known that proteins are very stable during cold acclimation (Hojka et al. 2014)⁠⁠. It is, therefore, possible that the cell actively downregulates the proteases during cold shift.

To our knowledge, this is the first model analyzing temperature acclimation in transcription and translation. The influence of temperature on biological systems has been extensively studied in connection with the circadian clock, which exhibits temperature compensation. Circadian clocks consist of oscillatory systems. Models studying the temperature compensation in these systems mostly focus on explaining how oscillations can be maintained with a fixed frequency despite the influence of temperature on chemical reactions (Kurosawa and Iwasa 2005)⁠⁠. It has been shown that this can be achieved by balancing reactions through a positive and a negative feedback-loop (Ruoff 1992)⁠⁠. This temperature compensation has been modeled by using the Goodwin negative feedback oscillator, which balances stabilizing and destabilizing reactions. Interestingly, this modeling approach suggests that the length of the oscillation in the circadian clock depends on the turnover rate of mRNA and protein (Ruoff et al. 2007)⁠⁠, which agrees with the observations from our model and substantiates the observation that degradation is a possible regulation mechanism also in cold acclimation. ⁠

Our model is a simple four-reaction model. Nevertheless, we can explain and simulate the observed behavior and make predictions. The rates in this model are relative and the reduction of the rates after cold treatment are only theoretical and should be adapted to precise experimental data, as soon as it becomes available. It would be necessary to further analyze the behavior of mRNA levels and translation activity during cold acclimation in plants. With time-series data on mRNA levels in cold-shifted plants it would be possible to verify the hypothesis that mRNA levels are also largely controlled by degradation, in case they are also constant during cold acclimation. Data on translation activity during this process would allow for further evaluation of the hypothesis that protein degradation is the main control point for protein concentration during acclimation to lower temperatures. It would also be interesting to experimentally unravel the process by which mRNA and protein degradation are controlled to stabilize protein concentration.

Despite the shortcomings of the model, we can reproduce the behavior observed in the western blots. We are able to model a biologically meaningful system, which does not show a decreased concentration during cold acclimation, as was observed for the three proteins analyzed here. We can make testable predictions about possible modes of regulation of the protein level during cold acclimation, which require further experimental validation.

## Supplementary Information

Below is the link to the electronic supplementary material.

Supplementary file1 (PDF 41 kb)
