# Supplementary material for: Modeling indicates degradation of mRNA and protein as a potential regulation mechanisms during cold acclimation
Source: J Plant Res. 2021 Apr 23;134(4):873–83. doi: 10.1007/s10265-021-01294-4 (PMC8245363; doi:10.1007/s10265-021-01294-4)
Supplement: Supplementary file 1 — Supplementary file1 (PDF 41 kb) [file 10265_2021_1294_MOESM1_ESM.pdf]

Electronic supplementary materials

Title:

Modeling indicates degradation of mRNA and protein as a potential regulation mechanisms during cold acclimation

Authors:

Maria Krantz, Julia Legen, Yang Gao, Reimo Zoschke, Christian Schmitz-Linneweber, Edda Klipp

Journal:

Journal of Plant Research

Corresponding author:

Prof. Dr. Dr. h.c. Edda Klipp

Humboldt-Universität zu Berlin, Institute of Biology,

Theoretical Biophysics, 10099 Berlin, Germany

Tel: +49 30 2093 8698

Fax: +49 30 2093 8813

E-mail: edda.klipp@rz.hu-berlin.de

Content:

Model Code

Model ODEs

Model Code

This code can be executed in python using the Stimator package.

```
from stimator import read_model, solve

import numpy as np
import pandas as pd
import matplotlib.pyplot as plt

model1 = """

vin3: -> mRNA  ,  rate = k3
vin4: -> Protein  ,  rate = k4*mRNA

vout6: mRNA ->  ,  rate = k6*mRNA
vout7: Protein ->  ,  rate = k7*Protein

k3 = 0.25
k4 = 0.25
k6 = 0.5
k7 = 0.1

#k3 = 2.5
#k4 = 2.5
#k6 = 1
#k7 = 1

init: (mRNA = 2.5, Protein = 6.25)


!! Protein mRNA
"""

M = read_model(model1)

print M

S = M.solve(tf=500)
print S
#S = np.array(S)
#print S.shape
```

S.plot(show=True)

Model ODEs

The reactions in the model are as follows:

mRNA import: -> mRNA , rate = k1
Protein production: -> Protein , rate = k3*mRNA
mRNA degradation: mRNA -> , rate = k2*mRNA
Protein degradation: Protein -> , rate = k4*Protein

The ODEs, which follow from these reactions, are as follows:

$$\frac{d}{dt}[mRNA] = k1 - k2 \cdot mRNA$$

$$\frac{d}{dt}[Protein] = k3 \cdot mRNA - k4 \cdot Protein$$
